# Cliffs Used as Communal Roosts by Andean Condors Protect the Birds from Weather and Predators

**DOI:** 10.1371/journal.pone.0067304

**Published:** 2013-06-24

**Authors:** Sergio A. Lambertucci, Adriana Ruggiero

**Affiliations:** Laboratorio Ecotono, INIBIOMA (CONICET-Universidad Nacional del Comahue), Bariloche, Río Negro, Argentina; Universidade de São Paulo, Faculdade de Filosofia Ciências e Letras de Ribeirão Preto, Brazil

## Abstract

The quality and availability of resources influence the geographical distribution of species. Social species need safe places to rest, meet, exchange information and obtain thermoregulatory benefits, but those places may also serve other important functions that have been overlooked in research. We use a large soaring bird that roosts communally in cliffs, the Andean condor (*Vultur gryphus*), as a model species to elucidate whether roost locations serve as a refuge from adverse weather conditions (climatic refuge hypothesis, CRH), and/or from predators or anthropogenic disturbances (threats refuge hypothesis, TRH). The CRH predicts that communal roosts will face in the opposite direction from where storms originate, and will be located in climatically stable, low precipitation areas. The TRH predicts that communal roosts will be large, poorly accessible cliffs, located far from human-made constructions. We surveyed cliffs used as communal roosts by condors in northwestern Patagonia, and compared them with alternative non-roosting cliffs to test these predictions at local and regional scales. We conclude that communal roosting places provide refuge against climate and disturbances such as, for instance, the threats of predators (including humans). Thus, it is not only the benefits gained from being aggregated per se, but the characteristics of the place selected for roosting that may both be essential for the survival of the species. This should be considered in management and conservation plans given the current scenario of global climate change and the increase in environmental disturbances.

## Introduction

The geographical distribution of a species is strongly influenced by resource availability. The quality of available resources can vary due to intrinsic characteristics as well as the ecological requirements of the species that use them [1]. For birds, location of food, nesting and roosting sites can be essential [2]. Particularly, for social species it is important to access safe places to rest, meet, exchange information or obtain thermoregulatory benefits [3]. Knowledge about how a species selects this resource at different geographical scales can provide clues to the ecological aspects associated with habitat use and behaviour, and can be useful to establish management strategies [4]–[6]. Moreover, this information can be used to analyse the potential habitat for a species, and to understand behaviours from an ecological and evolutionary point of view [2], [4]–[5].

Communal roosting is widely distributed among animals, and some of the proposed benefits of aggregation include the exchange of information for finding food, mate acquisition, and thermoregulatory purposes [3], [7]–[9]. There are a great number of studies devoted to the analysis of communal roosting behaviour in association with feeding habits [7], [10]–[12]. The potential advantages of communal roosting behaviour, unassociated with feeding behaviour, have been less studied (but see [8], [10], [13]). Moreover, the physical and environmental characteristics that may influence the selection of communal roosting places have also been rather overlooked [3].

Many species, including several raptors, use rocky cliffs as roosts, to rest or hunt [7], [14]. In particular, large soaring birds depend on places that provide refuge from predators (i.e., safe places), and allow them to take off easily, among other requirements [14], [15]. Hence, cliffs can be useful to analyse other potential advantages of communal roosting behaviour beyond its association with feeding habits. In particular, Andean condors (*Vultur gryphus*) roost communally in cliffs different from those in which they choose to nest (more detailed information is given in Methods, Study species) [16], [17].

The distribution and the aggregation patterns of condors in roosting places might be limited by the occurrence of roosts offering sun or protection, among other characteristics [16], [18]. Here, we proposed that communal roosts may serve as refuges from adverse weather conditions (“Climate Refuge Hypothesis”, CRH). In northwestern Patagonia (Argentina), the climate is cold-temperate, seasonally variable in terms of temperature, wind and precipitation, and adverse weather (strong winds, snow, etc.) comes mainly from the west-northwest [19]. Large flying birds are strongly limited by weather conditions, and avoid flying on rainy days [20]. The CRH predicts that: (1) On a regional scale, Andean condor communal roosts will be located in places with favourable weather conditions. Thus, in northwestern Patagonia (Argentina), communal roosts will be associated with warmer temperatures, lower variability in temperature and lower precipitation compared with cliffs not used for roosting. (2) At a local scale, where regional weather conditions are similar, the cliffs used for communal roosting will face in the direction opposite to that of predominant yearly winds, snow and rain.

Rocky cliffs can also provide a safe environment from potential predators [14], [21], and this could favour the evolution of the formation of communal roosts in birds [3], [10]. Condors face risks mainly when they are on the ground (e.g., eating), where they are very cautious [22]–[24], and also when they are roosting. At present, Pumas (*Puma concolor*), foxes (*Dusicyon* sp.), dogs (*Canis familiaris*), and humans could be perceived as a threat to condors during roosting [14], [25], [26]. Thus, if communal roosts offer refuge from threats posed by predation or anthropogenic disturbances (“Threats Refuge Hypothesis”, TRH), we predict that they will be in cliffs inaccessible to terrestrial predators and humans. To test this hypothesis at a local scale, we compared the geomorphologic characteristics of communal roosts with closed alternative non-roosting cliffs. On a regional scale, we predicted that if humans are perceived as a threat, cliffs used as communal roosts will be farther from towns, rural houses or roads, than alternative cliffs.

## Methods

### Study Species

The Andean condor is a Near Threatened large scavenger inhabiting the Andes mountain ranges in South America, and has suffered important retractions in several areas of its original geographical distribution [27]. Current populations are rare and reach maximum numbers in the southern area of the species’ distribution range [28]. The Andean condor does not breed communally but uses communal roosts to overnight [16]. Their nests may be located some hundreds of meters away in the areas surrounding their communal roosts, but can commonly be found as far as several kilometres away [16]–[17], [26]. Breeding adults frequently roost in the vicinities of the nest, but those roost-sites are used only by one individual rather than communal. Aggregations of individuals in the communal roosts may be more or less dispersed depending on the size of cliffs. Some of those shelves allow for the aggregations of dozens of individuals and others are small caves that can be used by one individual each. All sexes and age classes are represented in the communal roosts [18], [28], [29].

### Study Area

The study was carried out in a cold-temperate region in north-western Patagonia (Argentina) (ca. 40°–42°S and 70°–72°W). The Andes to the west act as a barrier to westerly winds at these latitudes, which produce variable weather conditions [19], and the predominance of winds blow from the west-northwest. The air masses from the Pacific Ocean are driven up and over the Andean mountain ranges causing the air to lose much of its moisture as precipitation on the Chilean side. Upon reaching the leeward side of the Andes the dry air descends and picks up any available moisture from the landscape below. This produces a high west-east gradient of precipitation in Argentina, where precipitation declines exponentially with distance from the Andes [19].

Towards the east, the Patagonian landscape is predominated by plains and erosive forms that have resulted in a number of outcrops serving as distinguishing characteristics of the local landscape. The number of roads and cars, and human density in the study area are low: 0.06 km of road/km^2^, from about 100 vehicles/day on gravel roads to 1550 vehicles/day on the national roads, and <0.6 inhabitants/km^2^ [23].

### Study Design

#### Choice of communal roosts and alternative cliffs

We surveyed the south of Neuquén and the northwest of the Río Negro provinces in Patagonia looking for Andean condor communal roosts based on information from local people and researchers, and previous data on roost location [16], [28]. Permission to study cliffs used by condors as communal roosts were provided by Dirección de Fauna Silvestre de Río Negro, the Argentine National Park Administration, and the owners and managers of local farms. We did not collect or manipulate birds in this study.

Proximity to food sources is a potentially important factor that may influence the presence of communal roosts in several species [7], [11], [12]. However, condors have large home ranges and can fly more than two hundred kilometres per day in order to find food sources [30] (Lambertucci et al. unpubl. data). Moreover, the same bird can move between communal roosts over a period of days [28], [31]. Nonetheless, to minimise the potential effect of food availability on our results we selected a geographically restricted study area that condors are able to cross in a few hours [30], and where the spatial variation of food resources is low (see below).

#### Data on a regional scale

We mapped a total of 29 communal roosts, within a rectangular study area of 150 km north-south×90 km west-east, to analyse the ecological and geomorphological determinants of the occurrence of communal roosts at a regional scale. We generated 29 random geographic coordinates within the same area using the “animal movement” extension of ArcView v3.3 [32]. These points were imported into Google Earth (http://earth.google.es/) to select the closest cliff to each random location. Then, we verified in the field that the location corresponded to a cliff not used by condors (i.e., no birds or faeces on the shelves). We estimated the same geomorphological climatic and anthropogenic variables for cliffs used and not used as communal roosts (see Table 1).

**Table 1:** Variables measured in cliffs used as communal roosts by the Andean condor (*Vultur gryphus*) and in alternative cliffs (not used for roosting) in the NW of Patagonia, Argentina.

Variable	Description
*Measures taken at the cliff*	
Aspect of the cliff (1,2)	Angular aspect of the cliff measured with a compass, estimated in degrees around the middle of the outcrop in the area with greatest number of shelves.
Altitude at the top	Altitude above sea level at the highest point of the cliff, measured with an altimeter. When the top could not be accessed, we used a clinometer and GPS.
Cliff height (1,2)	Difference between the altitude at the base of the cliff and the mean between the maximum and minimum altitude of the top.
Cliff width (1,2)	Linear distance between the lateral ends of the cliff. Measuring the coordinates of each extreme (with GPS) and calculating the distance between the points.
Floor-shelf distance (1,2)	Distance from the lowest shelf to the floor
Top-shelf distance (1,2)	Distance from the highest shelf to the top of the cliff.
Accessibility (1,2)	Accessibility to humans or possible terrestrial predator by foot, categorized as: high (3), medium (2) or low (1). We calculated the accessibility based on the quantity of shelves than could be reached by a terrestrial predator (e.g., puma (*Puma concolor*), fox (*Pseudalopex* spp.), ferret (*Galictis cuja*), etc.). We considered a *Low accessibility* (1); when >70% of the shelves were inaccessible; *Medium accessibility* (2): when around half of the shelves were inaccessible (40–70%); and *High accessibility* (3): when only <40% of the shelves were inaccessible.
*Measures taken around the cliff*	
Distance to building (1,2)	Distance from the cliff to the closest human construction measured in the field and by satellite images. Measured variables were: distance to edifice (house or farm) and distance to town (village or city). At a local scale, we only analysed the distance to the closest building since the distances between the communal roosts and other cliffs was low and almost invariable (see Methods).
Distance to road (1,2)	Distance from the cliff to the closest road measured from satellite images.
Annual mean temperature (BIO1) (2)	Annual mean temperature measured over a year^1^
Mean diurnal range (BIO2) (2)	Mean diurnal range (mean of monthly (max temp - min temp)) 1
Isothermality (BIO3) (2)	Isothermality (mean diurnal range (mean of monthly (max temp - min temp))/temperature annual range (max temperature of warmest month- min temperature of coldest month) (*100) 1
Annual precipitation (BIO12) (2)	Amount of precipitation over a year^1^
Precipitation seasonality (BIO15) (2)	Precipitation seasonality (coefficient of variation of the precipitation)^1^

(1) Variables used at local scale, (2) variables used at regional scale.

1 Obtained from WorldClim (www.worldclim.org), a digital global database that provides information on climate variables at a spatial resolution of ca. 1 km^2^ [33].

#### Data on a local scale

We selected 24 communal roosts at the centre of our study area out of the total 29 communal roosts mapped within the region. We walked along the four cardinal directions looking for the closest cliffs with apparently similar topographical and environmental characteristics to the central cliff used as a communal roost, but with no indication of use. We selected 3 or 4 of those alternative cliffs, depending on their availability and accessibility to the research team. These locations were also characterized in situ by the same set of variables estimated for communal roosts (see variables in Table 1). In total, we surveyed 109 cliffs: 24 communal roosts, and 85 alternative cliffs.

#### Climatic variables

We used the extension Grid Analyst v1.1 in ArcView v. 3.3. (ESRI, CA, USA) to assign values of five climatic variables to the geographic coordinates of each cliff based on the WorldClim digital database at a spatial resolution of 1 km^2^ (Hijmans *et al.* [33], available http://www.worldclim.org/current): 1) mean annual temperature, 2) mean annual precipitation, 3) diurnal mean range in temperature, 4) isothermality (diurnal mean range of temperature/annual range of temperature), and 5) seasonality of precipitation (Table 1).

We estimated the angular direction of the wind within our study region from records of wind direction registered by a weather station located in the town of Bariloche (41°07′04′′S–71°24′39′′W). This station registered 60961 records in 2007.

#### Geomorphologic variables and altitude

Each cliff was characterized in the field by aspect, height (m), width (m), the top-floor altitude of the cliff (m above sea level), distances from borders to shelves and accessibility of the cliff (Table 1).

#### Anthropogenic variables

Distances to the closest human buildings were included as indicators of anthropogenic disturbance. We distinguished between living places (houses, farms, towns) and roads (Table 1). At a local scale, we recorded the distance from each communal roost to the first building without any discrimination. Because the distances between communal roosts and alternative cliffs were short (mean distance between communal roosts and alternative cliffs was ca. 1.3 km) it can be assumed that they were at the same distance from distant built-up areas (towns). At a regional scale, we separately estimated the distances to the closest human building (house or farm) and to the closest built-up area (village or town; Table 1).

### Data Analysis

First, we compared the environmental attributes between communal roosts and alternative cliffs using non-parametric statistics [34]. We then applied logistic regressions [35] to determine the variables that better explained the use of a cliff for communal roosting, based on environmental predictors estimated at both local and regional scales; we included as a positive response (1) the communal roosts, and as a negative (0), the alternative roosts (Table 1). Variables were combined in the same statistical model provided they had a correlation of r <0.6 to reduce the problem of multicolinearity (see Table S1 in File S1).

We employed a multi-model selection approach [36] that involved an exhaustive search of all possible (single and multiple) logistic models to account for the presence of communal roosts. We included every possible combination of (low correlated, r<0.60) variables for each subset of predictors representing geomorphology, weather conditions, and anthropogenic disturbance. We used the Akaike’s Information Criterion (AIC, [35]), to find the best ecological model supported by our data among all possible models. For each group of predictors, the model with the lower AIC was selected as that model best supported by our data. Models with Δ_AIC_<2 from the best supported model were considered equivalents [36]. When several models had a Δ_AIC_<2, from the best models we selected the most parsimonious (i.e., with the lower number of variables). We combined the effects of variables from each of the best (geomorphologic, anthropogenic and climatic) models, and performed an exhaustive search of the “best final model” based on AIC. We used the McFadden’s rho test as a measure of the variation explained by the best final model. The McFadden`s rho is similar to the coefficient of determination (r^2^) of a linear regression, although it shows lower values. Values of McFadden’s rho between 0.2 and 0.4 are satisfactory [36]. We considered a coefficient estimate to be statistically significant when it was more than two standard errors away from zero [37].

The autocorrelation of variables across the geographic space is an inherent property of most ecological data, which often complicates the statistical testing of hypotheses by standard methods of analysis because it can inflate type I error rates, and may result in model instability [38]. We used SAM v. 4.0 [39] for previous analyses and to evaluate the effects of spatial structuring of variables on the performance of our environmental models. SAM v.4.0 allows the elaboration of a spatial correlogram using Moran’s (I) coefficient to describe the magnitude of spatial autocorrelation of variables for different distance classes. This coefficient range from −1 (maximal negative spatial autocorrelation) to +1 (maximal positive spatial autocorrelation), and values close to zero indicate no spatial correlation. We checked the adequacy of each environmental model through the examination of patterns of spatial autocorrelation in the residuals. Independent of the pattern of spatial autocorrelation in the original (predictors and response) variables, if no spatial autocorrelation is found in the residuals after including environmental predictors in the statistical model, then it can be concluded that the model has taken into account all spatial structure in the original data, and there is no statistical bias in the overall statistical analysis [38].

Finally, we applied circular statistics [40] to compare the aspect of roosts and alternative cliffs. We used the Rayleigh test to determine if the mean aspect angle, i.e. averaged over all roosts, differed from a random distribution around the 360° [41]. We applied the Watson U^2^ test [41] to look for significant differences between the aspect of communal roosts and alternative cliffs, and to compare the aspect of communal roosts with respect to angular wind direction.

#### Test of the effect of site and food availability as potential confounding variables

Since at a local scale communal roosts and alternative cliffs are located close to each other, we applied a logistic regression with “site” as the only explicative variable; given that there was no significant effect of site (Wald = 0.499, P = 0.99), we did not control for site effects in any subsequent analyses. We also evaluated the possible effect of food availability on the use of communal roosts. We must emphasize here that condors in our study area were able to come and go from any of the roosts studied [31] (Lambertucci et al. unpubl. data), hence it was reasonable to assume that food resources were not a limiting factor influencing the use of any cliff for roosting. Nonetheless, to confirm this assumption we selected a subset of 18 communal roosts and 17 alternative cliffs for which data on the density of livestock in the surroundings were available [42]. Given that we did not find evidence that the presence of communal roosts was associated with the density of livestock (Logistic Regression Model including only livestock abundance as an explanatory variable, Wald = 0.097; P = 0.755), food availability was not included in any subsequent statistical analyses.

## Results

### Overall Differences between Communal Roosts and Alternative Cliffs

Communal roosts were larger and less accessible, with shelves more distant from the borders of the cliff, than alternative cliffs. However, both were at similar altitude above sea level and at similar distances to the closest building or road (Table 2).

**Table 2:** Environmental and geomorphological characteristics of cliffs studied.

Variables	Communal roosts		Alternative cliffs		Mann-Whitney U-Test	
	n = 24 (± DS)		n = 85 (± DS)			
Aspect (°, degrees)	108,9	(63,2)	AD			
Accessibility (1–3)*	1,2	(0,48)	2,2	(0,77)	U = 310	P<0.001
Cliff width (m)	341,3	(178,0)	145,5	(122,0)	U = 279	P<0.001
Cliff height (m)	105,9	(56,2)	46,7	(37,5)	U = 282	P<0.001
Altitude at the top (msnm)	1267,3	(269,9)	1171	(229,2)	U = 764,	P = 0,061
Floor-shelf distance (m)	23	(18,0)	6,8	(7,2)	U = 305	P<0.001
Top-shelf distance (m)	15	(12,7)	5,2	(7,4)	U = 390	P<0.001
Distance to building (m)	2531,7	(1210,5)	2479,4	(1317,4)	U = 956	P = 0.468
Distance to road (m)	1268,8	(1162,1)	1273,1	(1079,6)	U = 1009	P = 0.939

Mean values (± Standard deviation, SD) from different variables measured in Andean condor communal roosts and cliffs not used as communal roosts (alternative cliffs) and their statistical comparisons (Mann-Whitney U-test).

* categorical variable (1 lower- 3 higher accessibility). AD =  all directions.

### Factors Associated with the Presence of Communal Roosts at a Local Scale

**Figure 1:**
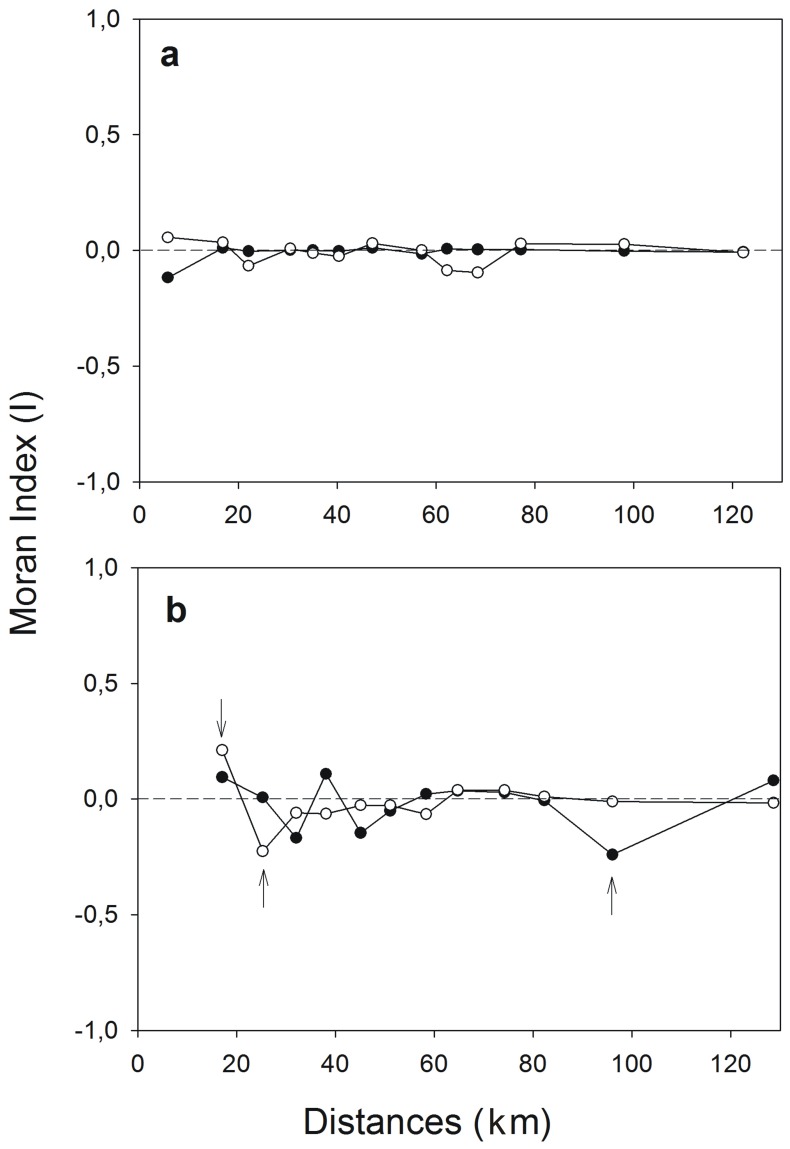
Spatial correlograms. Moran’s index to detect the presence of spatial autocorrelation (arrows = I<0.05) in the response variable “presence of cliffs” (black dots) and in the residuals (open dots) from the best logistic model fitted at a local (a), and at a regional scale (b) (see Methods).

The model including geomorphological variables only was the best supported by our data (Table 3; Table S2 in File S1). The presence of communal roosts was associated with wide cliffs of low accessibility, with a long distance (a mean of 23 m) from the floor to the lowest shelf, and eastern aspect. Cliff height, distance from the top to the highest shelf, and distances to roads and buildings had no significant association with the presence of communal roosts at a local spatial scale (Table 3). There was a very low (I = −0.12), negative spatial autocorrelation in the original response variable at the lowest distance class (<5 km); the lack of spatial autocorrelation in the residuals from the best geomorphological model (i.e., for all distant classes I<0.1; Fig. 1a) suggested that our statistical models were robust and adequate to account for the spatial variation in the use of roosts at a local scale.

**Table 3:** Models at a local scale that best distinguished between Andean condor communal roosts and alternative cliffs.

Model	Rho	AIC	Variables	Coefficient	Standard coefficient	Standard error
Anthropogenic	<0.001	118.89	Intercept	−1.343	0	0.505
			Distance to building	<0.001	0.097	<0.001
Geomorphologic ( = Mixed)	**0.709**	**43.46**	Intercept	2.719	0	1.687
			Aspect	−4.905	−5.906	**1.830**
			Accesibility	−1.944	−3.918	**0.660**
			Cliff width	0.013	5.083	**0.005**
			Floor-shelf distance	0.208	6.211	**0.074**

Best final logistic regression models at a local scale that included variables representing anthropogenic disturbances, and geomorphology that best distinguished between 24 Andean condor communal roosts and 85 alternative cliffs selected around the roost. We included the value of McFadden’s Rho-Squared (Rho) and Akaike’s Information Criterion (AIC). Numbers in bold are statistically different (i.e., they are more than 2 standard errors away from zero).

### Factors Associated with the Presence of Communal Roosts at a Regional Scale

A mixed model that combined climatic and geomorphological variables was the best supported by our data to account for the presence of communal roosts at a regional scale (Table 4; Table S3 in File S1). Communal roosts were located in places with high isothermality and low precipitation; they faced toward the east and had a high floor-shelf distance. Although the distance to town was an important predictor of the presence of communal roosts in the best anthropogenic model, it was not in the final mixed model (Table 4). The spatial autocorrelation present in the response variable near 100 km (I = −0.24) approached zero in the residuals from the best ecological model (Fig. 1b). This suggests that the environmental predictors adequately accounted for the use of communal roosts at large geographic scales within the spatial extent of our study. At the shortest distance classes (<30 km), spatial autocorrelation remained in the residuals after model fit, although it was low (I<0.23) and hence, this suggested that the effect of spatial autocorrelation did not severely influence the performance of our statistical model (Fig. 1b).

**Table 4:** Models at a regional scale that best distinguished between Andean condor communal roosts and alternative cliffs.

Model	Rho	AIC	Variables	Coefficient	Standard coefficient	Estándar error
Climatic	0.283	63.64	Intercept	−27.61	0	12.109
			Isothermality	6.188	4.363	**2.52**
			Anual Precipitation	−0.005	−1.581	**0.002**
Anthropogenic	0.099	78.41	Intercept	−0,813	0	0,688
			Distance to town	0,090	1,392	**0,039**
Geomorphologic	0.451	52.13	Intercept	−0,371	0	1,409
			Aspect	−1,959	−1,883	**0,853**
			Cliff width	0,006	2,276	**0,002**
			Floor-shelf distance	0,106	3,625	**0,050**
Mixed	**0.648**	**38.29**	Intercept	−10.909	0	12.581
			Aspect	−3.802	−3.655	**1.307**
			Floor-shelf distance	0.243	8.309	**0.101**
			Isothermality	4.5	3.173	**2.621**
			Annual precipitation	−0.014	−4.72	**0.005**

Final logistic regression models built at a regional scale by a group of variables (climatic, anthropogenic, and geomorphologic) and the mixed model that best distinguished the 29 Andean condor communal roosts from the 29 alternative cliffs. We included the value of McFadden’s Rho-Squared (Rho) and Akaike’s Information. Criterion (AIC). Numbers in bold are statistically different (i.e., they are more than two standard errors away from zero).

### Cliff Aspect and Direction of Winds

**Figure 2:**
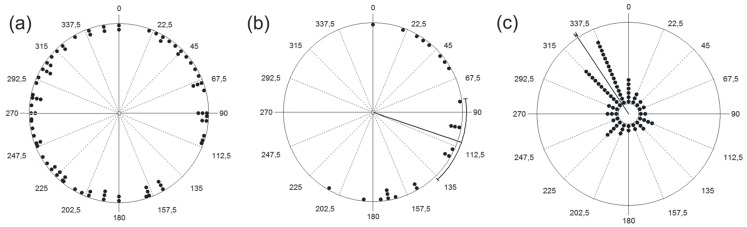
Distribution of the aspect of condor communal roosts, alternative cliffs and wind direction. (a) Circular plots showing the distribution of aspects of cliffs (black points represent cliffs) not used as communal roosts in the northwest of Patagonia; (b) Aspect of 24 Andean condor (*Vultur gryphus*) communal roosts. Points in the radius of the circle correspond to the number of roosts. (c) Wind direction throughout 2007 in the NW of Patagonia. Each point corresponds to ca. 870 data points registered by the weather station. In every graph we indicate the values of the angles (numbers outside the circle), and for (b) and (c), the mean aspect and its confidence interval (CI 95%) with a thin black line.

The aspect of non-roosting cliffs in northwestern Patagonia was randomly distributed (Rayleigh, N = 82; Z = 1.10; P = 0.33; Fig. 2a). In contrast, cliffs aspect of condor communal roosts was not randomly distributed (Rayleigh, N = 24; Z = 7.11; P<0.001), and the mean angle was orientated toward the east-southeast at 108.9°N (r = 0.544; CI (95%) = 81.6°–136.1°; median = 107.5°; Fig. 2b). Consequently, the aspect of cliffs used as communal roosts and alternative cliffs differed (Watson, U^2^ = 0.42; P<0.001).

The wind direction was not randomly distributed in north-western Patagonia (Rayleigh, N = 60961; Z = 12027.2; P<0.0001), and its mean angle of direction at 326°N (r = 0.444; CI (95%) = 325.6°–327.0°; median = 337.5°; Fig. 2c) is opposite to that of the aspect of communal roosts (Fig. 2b).

## Discussion

We found that the use of a cliff for roosting may involve a twofold selection process for sites that offer shelter against natural or anthropogenic threats while also serving as a refuge from unfavourable weather conditions at both local and regional scales. Thus, the proximity to food sources or other benefits of aggregation are not unique in their role in determining the use of a place as a communal roost as is often suggested [3], [7], [8], [11]. At a local scale, we showed that the geomorphology of the cliff is important in distinguishing between roosting cliffs and those not used for roosting, which agrees with studies in other species (e.g., for a mammal: [43], for a bird: [44]). At a regional scale, cliffs used for communal roosting are located in places with low precipitation and more stable temperatures, which suggest that climatic variables may play an important role in the use of sites to overnight. The lack or low levels of spatial autocorrelation that remained in the residuals after the fit of our statistical models suggests that our results were robust and not severely influenced by the presence of spatial autocorrelation in our original data [38]. As far as the local spatial scale of our analysis was concerned, the density of livestock did not account for the presence of communal roosts, suggesting that it is unlikely that proximity to food resources had been an important factor underlying the use of particular cliffs for communal roosting by the Andean condor.

We showed that climate was an important factor to account for the presence of communal roosts at a regional scale, thus supporting the climatic refuge hypothesis (CRH). Weather conditions are known to be very important in modulating the behaviour and habitat selection of many species [44]–[47]. The location of communal roosts coincides with sites of lower precipitation and lower variation in temperature rather than alternative cliffs. Moreover, condor communal roosts were typically located in large cliffs that faced in the direction opposite to the predominant winds. Cliff aspect may also affect the microclimatic conditions at the roosting place, as it influences the quantity of radiation (daily, seasonal and latitudinal), temperature, wind and precipitation received at a local scale [21]. Cliff facing in the direction opposite to the wind can accumulate lower amounts of snow [48], and are frequently selected by different raptor species [21], [49].

It has been proposed that aggregated roosting imparts thermoregulatory benefits [3], [13]. Although condors may benefit from aggregation in close proximity to conspecifics in the communal roosts (not evaluated in this work), our results suggest that the roosts themselves may provide thermoregulatory benefits due to physical and geographic characteristics associated with morphology, aspect and geographical location. Specifically, large cliffs, facing opposite to the predominant winds, and located in places with low temperature variability and low precipitation, may be suitable under adverse weather conditions [21], when communally roosting birds could be injured [50]. Therefore, cliffs may be important climate refuges for condors, and thus it seems reasonable to predict that if future global warming alters the predominant weather conditions within the region [51], this might modify their quality as refuges.

On the other hand, cliffs selected as communal roosts were large, poorly accessible outcrops, which supports the idea that they may be good refuges from possible threats (predation or anthropogenic disturbances, TRH). The extent to which cliffs are accessible to predators seems to be relevant for communal roosting. We found that distance from the floor to the lowest shelf was associated with the presence of a communal roost. Cliffs with lower shelves, at short distances from the floor, could be more accessible to species that can be considered as potential predators of condors (e.g., pumas or humans) and, thus, dangerous to roosting birds. In contrast, the distance from the highest shelf to the top of the cliff was not important. This is not surprising given that the top of the cliffs are inaccessible for terrestrial species that may be considered as a potential predator. The dilution effect hypothesis proposes that the gathering of many individuals in communal roosts may reduce the individual depredation risk [52] with the centre of the roost being the safest [10]. Our study favors the idea that condors use large and inaccessible cliffs to diminish predation and disturbance, but other explanations could also affect roost selection. For instance, large-heavy birds such as condors are limited in their capacity to fly [14], [15], [53], which presents the possibility that these birds use large cliffs, with high shelves, to help them take off. Moreover, communal roosts oriented toward the sunrise (east) favor thermal lift and benefit soaring flight. In the southern hemisphere, east and north-east facing cliffs are warmer, mainly during the morning, which could be important to increase thermal activity more so than would occur in west-facing cliffs and to warm the birds for an easier transition in leaving the location. On the other hand, the amount of overhang at the top of the cliff and above the shelves could also affect the terrestrial radiation useful during nighttime thermal conditions. None of these hypotheses are mutually exclusive, and deserve detailed consideration in future studies.

Although cliffs used as communal roosts were in general far from buildings, distance to town was not important in accounting for the presence of a communal roost in the presence of climatic and geomorphological variables. At present, the number of roads, car traffic and extent of human population is still low in our study area [23], which might explain why variables representing human disturbance were less explicative in our study. Other large avian scavengers are known to avoid human constructions selectively (California condor [25], [54] Old world vultures [55], [56]); sometimes they can be tolerant to human constructions when they find a location appropriate, for instance, to rest or breed [57]. However, this can pose other problems such as the introduction of competitor species, furtive hunting, and nesting failure [24], [26], [58], [59]. Thus, as a precautionary principle, changes in land use should be considered and anthropogenic disturbances included in future evaluations of hypotheses on the use of communal roosts.

Previous studies that test hypotheses on communal roosting behaviour were mainly focused on the species’ decisions once the birds selected a particular roost (eg., [8], [9], [13]), but not on the identification of environmental characteristics that might be involved in the decision of which places were used to roost. Individual birds that visit a communal roost may be independently searching for places with particular characteristics [3], and we found that condor communal roosts typically provide protection against unfavourable weather conditions, and refuge from possible threats, supporting both the climatic and the threats refuge hypotheses. Warm, climatically protected, inaccessible roosting places can reduce the possible survival costs of overnighting in very cold places, or the mortality risk of being in places highly exposed to predators or human disturbances [3], [13], [18]. Therefore, communal roosts can be a valuable resource and deserve special attention in the development of long-term conservation practices for condors, and likely for other species as well [3], [9], [29], under the current scenario of climatic change and increases in anthropogenic disturbances.

## Supporting Information

File S1 Table S1, Correlations between anthropogenic, geomorphological and climatic variables included in the statistical models. Table S2, Exhaustive search of best models to account for the presence of communal roosts at a local scale. Table S3, Exhaustive search of best models to account for the presence of communal roosts at a regional scale.
(DOC)
