# Supplementary material for: Cliffs Used as Communal Roosts by Andean Condors Protect the Birds from Weather and Predators
Source: PLoS One. 2013 Jun 24;8(6):e67304. doi: 10.1371/journal.pone.0067304 (PMC3691149; doi:10.1371/journal.pone.0067304)
Supplement: File S1 — Table S1, Correlations between anthropogenic, geomorphological and climatic variables included in the statistical models. Table S2, Exhaustive search of best models to account for the presence of communal roosts at a local scale. Table S3, Exhaustive search of best models to account for the presence of communal roosts at a regional scale. (DOC) [file pone.0067304.s001.doc]

File S1

Supporting Information for:

**Cliffs used as communal roosts by the Andean condor: are they refuges against bad weather conditions and threats?** Sergio A. Lambertucci* and Adriana Ruggiero

This material includes:

- Correlations between variables
- Anthropic and morphologic models for local scale
- Anthropic, climatic, morphologic and mixed models for regional scale

Table S1. Correlations between anthropogenic, geomorphological and climatic variables to be included in the statistical models. Variables were combined in the same statistical model provided they have a correlation r < 0.6 to reduce the problem of multicolinearity. Correlations higher than 0.6 are indicated in bold.

1. Correlations at local scale

|  | Aspect | Accesibility | Cliff wide | Cliff height | Floor-shelf distance | Top-shelf distance | Distance to building | Distance to road |
| --- | --- | --- | --- | --- | --- | --- | --- | --- |
| Aspect | 1,00 | 0,13 | -0,17 | -0,16 | -0,20 | -0,13 | -0,06 | -0,05 |
| Accesibility |  | 1,00 | -0,28 | -0,32 | -0,37 | -0,36 | -0,08 | 0,10 |
| Cliff wide |  |  | 1,00 | 0,50 | 0,38 | 0,20 | 0,13 | 0,05 |
| Cliff height |  |  |  | 1,00 | 0,53 | 0,41 | -0,05 | -0,03 |
| Floor-shelf distance |  |  |  |  | 1,00 | 0,59 | 0,11 | 0,05 |
| Top-shelf distance |  |  |  |  |  | 1,00 | -0,06 | -0,13 |
| Distance to building |  |  |  |  |  |  | 1,00 | 0,09 |
| Distance to road |  |  |  |  |  |  |  | 1,00 |

1. Correlations at regional scale

|  | Accesibility | Cliff wide | Cliff height | Floor-shelf distance | Top-shelf distance | Distance to building | Distance to road | Distance to town | Annual mean temperature | Mean diurnal range | Isothermality | Annual precipitation | Precipitation seasonality |
| --- | --- | --- | --- | --- | --- | --- | --- | --- | --- | --- | --- | --- | --- |
| Accesibility | 1,00 | -0,09 | -0,22 | -0,27 | -0,22 | -0,05 | 0,02 | -0,17 | 0,06 | -0,01 | -0,05 | 0,01 | 0,07 |
| Cliff wide |  | 1,00 | 0,45 | 0,23 | 0,08 | -0,09 | -0,21 | 0,14 | 0,21 | 0,26 | 0,33 | -0,21 | 0,20 |
| Cliff height |  |  | 1,00 | 0,44 | 0,22 | 0,07 | -0,18 | -0,06 | -0,01 | -0,30 | 0,20 | 0,23 | -0,10 |
| Floor-shelf distance |  |  |  | 1,00 | **0,73** | 0,11 | 0,17 | 0,12 | -0,22 | -0,10 | 0,36 | 0,14 | -0,21 |
| Top-shelf distance |  |  |  |  | 1,00 | 0,11 | 0,16 | 0,10 | -0,12 | -0,09 | 0,20 | 0,13 | -0,14 |
| Distance to building |  |  |  |  |  | 1,00 | 0,33 | -0,07 | -0,15 | -0,27 | -0,01 | 0,30 | -0,27 |
| Distance to road |  |  |  |  |  |  | 1,00 | -0,01 | -0,50 | -0,23 | -0,13 | 0,22 | -0,40 |
| Distance to town |  |  |  |  |  |  |  | 1,00 | 0,01 | 0,25 | 0,26 | -0,26 | 0,07 |
| Annual mean temperature |  |  |  |  |  |  |  |  | 1,00 | 0,47 | 0,18 | -0,46 | **0,81** |
| Mean diurnal range |  |  |  |  |  |  |  |  |  | 1,00 | 0,30 | **-0,94** | **0,76** |
| Isothermality |  |  |  |  |  |  |  |  |  |  | 1,00 | -0,22 | 0,17 |
| Annual precipitation |  |  |  |  |  |  |  |  |  |  |  | 1,00 | **-0,82** |
| Precipitation seasonality |  |  |  |  |  |  |  |  |  |  |  |  | 1,00 |

Table S2. Exhaustive search of best models to account for the presence of communal roosts at local scale. The Akaike information criterion (AIC) was used to select the most likely model supported by our data. Models with differences in the Akaike value (ΔAIC) less than 2 from the best model (indicated in bold) were equally likely. Final selected model is in grey. DF= Degree of freedom.

1. Search of the best anthropogenic disturbance model.

| Variable 1 | Variable 2 | DF | AIC | ΔAIC |
| --- | --- | --- | --- | --- |
| Distance to building |  | 1 | 118.89 | **0.00** |
| Distance to road |  | 1 | 118.92 | **0.03** |
| Distance to building | Distance to road | 2 | 120.88 | **1.99** |

1. Search of the best geomorphological model.

| Variable 1 | Variable 2 | Variable 3 | Variable 4 | Variable 5 | Variable 6 | DF | AIC | ΔAIC |
| --- | --- | --- | --- | --- | --- | --- | --- | --- |
| Aspect | Accesibility | Cliff wide | Floor-shelf distance |  |  | 4 | 43,46 | **0,00** |
| Aspect | Accesibility | Cliff wide | Cliff height | Floor-shelf distance |  | 5 | 44,34 | **0,89** |
| Aspect | Accesibility | Cliff wide | Floor-shelf distance | Top-shelf distance |  | 5 | 45,29 | **1,83** |
| Aspect | Accesibility | Cliff wide | Cliff height | Floor-shelf distance | Top-shelf distance | 6 | 46,30 | > 2 |
| Aspect | Accesibility | Cliff wide | Cliff height | Top-shelf distance |  | 5 | 52,16 | > 2 |
| Aspect | Accesibility | Cliff wide | Cliff height |  |  | 4 | 52,34 | > 2 |
| Aspect | Accesibility | Cliff wide | Top-shelf distance |  |  | 4 | 53,56 | > 2 |
| Aspect | Accesibility | Cliff height | Floor-shelf distance |  |  | 4 | 53,74 | > 2 |
| Aspect | Cliff wide | Cliff height | Floor-shelf distance |  |  | 4 | 54,53 | > 2 |
| Aspect | Cliff wide | Floor-shelf distance |  |  |  | 3 | 54,71 | > 2 |
| Aspect | Cliff wide | Floor-shelf distance | Top-shelf distance |  |  | 4 | 55,00 | > 2 |
| Aspect | Accesibility | Cliff wide |  |  |  | 3 | 55,13 | > 2 |
| Aspect | Cliff wide | Cliff height | Floor-shelf distance | Top-shelf distance |  | 5 | 55,38 | > 2 |
| Aspect | Accesibility | Cliff height | Floor-shelf distance | Top-shelf distance |  | 5 | 55,73 | > 2 |
| Aspect | Accesibility | Floor-shelf distance |  |  |  | 3 | 56,34 | > 2 |
| Aspect | Accesibility | Cliff height |  |  |  | 3 | 57,40 | > 2 |
| Accesibility | Cliff wide | Cliff height | Floor-shelf distance |  |  | 4 | 57,52 | > 2 |
| Accesibility | Cliff wide | Floor-shelf distance |  |  |  | 3 | 57,92 | > 2 |
| Aspect | Accesibility | Floor-shelf distance | Top-shelf distance |  |  | 4 | 58,09 | > 2 |
| Aspect | Accesibility | Cliff height | Top-shelf distance |  |  | 4 | 58,26 | > 2 |
| Accesibility | Cliff wide | Cliff height | Floor-shelf distance | Top-shelf distance |  | 5 | 58,82 | > 2 |
| Accesibility | Cliff wide | Floor-shelf distance | Top-shelf distance |  |  | 4 | 58,90 | > 2 |
| Accesibility | Cliff wide | Cliff height | Top-shelf distance |  |  | 4 | 60,72 | > 2 |
| Accesibility | Cliff wide | Cliff height |  |  |  | 3 | 62,33 | > 2 |
| Aspect | Cliff wide | Cliff height | Top-shelf distance |  |  | 4 | 62,33 | > 2 |
| Accesibility | Cliff wide | Top-shelf distance |  |  |  | 3 | 63,02 | > 2 |
| Accesibility | Cliff height | Floor-shelf distance |  |  |  | 3 | 63,91 | > 2 |
| Aspect | Cliff wide | Top-shelf distance |  |  |  | 3 | 64,75 | > 2 |
| Aspect | Accesibility | Top-shelf distance |  |  |  | 3 | 65,07 | > 2 |
| Accesibility | Cliff height | Floor-shelf distance | Top-shelf distance |  |  | 4 | 65,80 | > 2 |
| Accesibility | Cliff wide |  |  |  |  | 2 | 66,62 | > 2 |
| Aspect | Cliff wide | Cliff height |  |  |  | 3 | 66,67 | > 2 |
| Aspect | Accesibility |  |  |  |  | 2 | 67,17 | > 2 |
| Aspect | Cliff height | Floor-shelf distance |  |  |  | 3 | 67,19 | > 2 |
| Accesibility | Cliff height |  |  |  |  | 2 | 67,28 | > 2 |
| Accesibility | Cliff height | Top-shelf distance |  |  |  | 3 | 67,40 | > 2 |
| Aspect | Cliff height | Floor-shelf distance | Top-shelf distance |  |  | 4 | 68,41 | > 2 |
| Accesibility | Floor-shelf distance |  |  |  |  | 2 | 68,72 | > 2 |
| Accesibility | Floor-shelf distance | Top-shelf distance |  |  |  | 3 | 70,37 | > 2 |
| Aspect | Cliff wide |  |  |  |  | 2 | 72,46 | > 2 |
| Cliff wide | Cliff height | Floor-shelf distance | Top-shelf distance |  |  | 4 | 72,90 | > 2 |
| Cliff wide | Floor-shelf distance | Top-shelf distance |  |  |  | 3 | 73,12 | > 2 |
| Cliff wide | Cliff height | Floor-shelf distance |  |  |  | 3 | 73,24 | > 2 |
| Aspect | Cliff height | Top-shelf distance |  |  |  | 3 | 73,68 | > 2 |
| Cliff wide | Floor-shelf distance |  |  |  |  | 2 | 74,03 | > 2 |
| Aspect | Floor-shelf distance |  |  |  |  | 2 | 74,14 | > 2 |
| Aspect | Floor-shelf distance | Top-shelf distance |  |  |  | 3 | 74,23 | > 2 |
| Cliff wide | Cliff height | Top-shelf distance |  |  |  | 3 | 76,72 | > 2 |
| Aspect | Cliff height |  |  |  |  | 2 | 76,97 | > 2 |
| Accesibility | Top-shelf distance |  |  |  |  | 2 | 77,65 | > 2 |
| Cliff wide | Top-shelf distance |  |  |  |  | 2 | 80,82 | > 2 |
| Cliff height | Floor-shelf distance |  |  |  |  | 2 | 81,83 | > 2 |
| Accesibility |  |  |  |  |  | 1 | 81,93 | > 2 |
| Cliff height | Floor-shelf distance | Top-shelf distance |  |  |  | 3 | 82,58 | > 2 |
| Cliff wide | Cliff height |  |  |  |  | 2 | 83,16 | > 2 |
| Aspect | Top-shelf distance |  |  |  |  | 2 | 85,64 | > 2 |
| Floor-shelf distance |  |  |  |  |  | 1 | 88,83 | > 2 |
| Floor-shelf distance | Top-shelf distance |  |  |  |  | 2 | 88,89 | > 2 |
| Cliff height | Top-shelf distance |  |  |  |  | 2 | 89,15 | > 2 |
| Cliff wide |  |  |  |  |  | 1 | 92,36 | > 2 |
| Cliff height |  |  |  |  |  | 1 | 93,86 | > 2 |
| Aspect |  |  |  |  |  | 1 | 97,87 | > 2 |
| Top-shelf distance |  |  |  |  |  | 1 | 102,27 | > 2 |

Table S3. Exhaustive search of best models to account for the presence of communal roosts at regional scale. The Akaike information criterion (AIC) was used to select the most likely model supported by our data. Models with differences in the Akaike value (ΔAIC) less than 2 from the best model (indicated in bold) were equally likely. Final selected model is in grey. DF= Degree of freedom.

1. Search of the best geomorphologic model.

| Variable 1 | Variable 2 | Variable 3 | Variable 4 | Variable 5 | Variable 6 | DF | AIC | ΔAIC |
| --- | --- | --- | --- | --- | --- | --- | --- | --- |
| Aspect | Cliff wide | Floor-shelf distance |  |  |  | 3 | 52.13 | **0.00** |
| Aspect | Accesibility | Cliff wide | Floor-shelf distance |  |  | 4 | 53.38 | **1.25** |
| Aspect | Cliff wide | Cliff height | Floor-shelf distance |  |  | 4 | 53.56 | **1.43** |
| Aspect | Cliff wide | Top-shelf distance |  |  |  | 3 | 54.19 | > 2 |
| Aspect | Accesibility | Cliff wide | Cliff height | Floor-shelf distance |  | 5 | 54.88 | > 2 |
| Accesibility | Cliff wide | Floor-shelf distance |  |  |  | 3 | 55.49 | > 2 |
| Aspect | Accesibility | Cliff wide | Top-shelf distance |  |  | 4 | 55.69 | > 2 |
| Cliff wide | Floor-shelf distance |  |  |  |  | 2 | 56.07 | > 2 |
| Aspect | Cliff wide | Cliff height | Top-shelf distance |  |  | 4 | 56.17 | > 2 |
| Aspect | Floor-shelf distance |  |  |  |  | 2 | 57.47 | > 2 |
| Accesibility | Cliff wide | Cliff height | Floor-shelf distance |  |  | 4 | 57.49 | > 2 |
| Aspect | Accesibility | Cliff wide | Cliff height | Top-shelf distance |  | 5 | 57.67 | > 2 |
| Cliff wide | Cliff height | Floor-shelf distance |  |  |  | 3 | 58.07 | > 2 |
| Accesibility | Cliff wide | Top-shelf distance |  |  |  | 3 | 58.45 | > 2 |
| Cliff wide | Top-shelf distance |  |  |  |  | 2 | 58.58 | > 2 |
| Aspect | Accesibility | Floor-shelf distance |  |  |  | 3 | 58.71 | > 2 |
| Aspect | Cliff wide |  |  |  |  | 2 | 58.75 | > 2 |
| Aspect | Accesibility | Cliff wide |  |  |  | 3 | 58.76 | > 2 |
| Aspect | Top-shelf distance |  |  |  |  | 2 | 59.19 | > 2 |
| Aspect | Cliff height | Floor-shelf distance |  |  |  | 3 | 59.44 | > 2 |
| Accesibility | Cliff wide | Cliff height | Top-shelf distance |  |  | 4 | 60.22 | > 2 |
| Aspect | Accesibility | Top-shelf distance |  |  |  | 3 | 60.29 | > 2 |
| Cliff wide | Cliff height | Top-shelf distance |  |  |  | 3 | 60.33 | > 2 |
| Aspect | Accesibility | Cliff height | Floor-shelf distance |  |  | 4 | 60.66 | > 2 |
| Aspect | Cliff wide | Cliff height |  |  |  | 3 | 60.74 | > 2 |
| Aspect | Accesibility | Cliff wide | Cliff height |  |  | 4 | 60.76 | > 2 |
| Aspect | Cliff height | Top-shelf distance |  |  |  | 3 | 60.88 | > 2 |
| Aspect | Accesibility | Cliff height | Top-shelf distance |  |  | 4 | 62.10 | > 2 |
| Accesibility | Cliff wide |  |  |  |  | 2 | 63.72 | > 2 |
| Accesibility | Cliff wide | Cliff height |  |  |  | 3 | 64.80 | > 2 |
| Aspect | Accesibility |  |  |  |  | 2 | 65.48 | > 2 |
| Aspect |  |  |  |  |  | 1 | 66.25 | > 2 |
| Aspect | Accesibility | Cliff height |  |  |  | 3 | 66.34 | > 2 |
| Accesibility | Floor-shelf distance |  |  |  |  | 2 | 66.36 | > 2 |
| Aspect | Cliff height |  |  |  |  | 2 | 66.37 | > 2 |
| Floor-shelf distance |  |  |  |  |  | 1 | 66.41 | > 2 |
| Cliff height | Floor-shelf distance |  |  |  |  | 2 | 67.11 | > 2 |
| Cliff wide |  |  |  |  |  | 1 | 67.11 | > 2 |
| Accesibility | Cliff height | Floor-shelf distance |  |  |  | 3 | 67.14 | > 2 |
| Cliff wide | Cliff height |  |  |  |  | 2 | 67.49 | > 2 |
| Cliff height | Top-shelf distance |  |  |  |  | 2 | 68.22 | > 2 |
| Accesibility | Cliff height | Top-shelf distance |  |  |  | 3 | 68.32 | > 2 |
| Accesibility | Top-shelf distance |  |  |  |  | 2 | 69.04 | > 2 |
| Top-shelf distance |  |  |  |  |  | 1 | 69.30 | > 2 |
| Accesibility | Cliff height |  |  |  |  | 2 | 73.22 | > 2 |
| Cliff height |  |  |  |  |  | 1 | 75.24 | > 2 |
| Accesibility |  |  |  |  |  | 1 | 78.09 | > 2 |

4

1. Search of the best anthropic model.

| Variable 1 | Variable 2 | Variable 3 | DF | AIC | DAIC |
| --- | --- | --- | --- | --- | --- |
| Distance to town |  |  | 1 | 78.41 | **0.0000** |
| Distance to road | Distance to town |  | 2 | 78.44 | **0.03** |
| Distance to building | Distance to road | Distance to town | 3 | 79.90 | **1.49** |
| Distance to building | Distance to town |  | 2 | 80.36 | **1.95** |
| Distance to road |  |  | 1 | 82.54 | > 2 |
| Distance to building | Distance to road |  | 2 | 84.27 | > 2 |
| Distance to building |  |  | 1 | 84.40 | > 2 |

1. Search of the best climatic model.

| Variable 1 | Variable 2 | Variable 3 | DF | AIC | ΔAIC |
| --- | --- | --- | --- | --- | --- |
| Annual mean temperature | Isothermality | Annual precipitation | 3 | 62.89 | **0.00** |
| Isothermality | Annual precipitation |  | 2 | 63.64 | **0.75** |
| Annual mean temperature | Isothermality |  | 2 | 64.83 | **1.94** |
| Isothermality |  |  | 1 | 67.17 | > 2 |
| Annual precipitation |  |  | 1 | 78.80 | > 2 |
| Annual mean temperature | Annual precipitation |  | 2 | 80.41 | > 2 |
| Annual mean temperature |  |  | 1 | 81.76 | > 2 |

1. Search of the best mixed model that combines geomorphological, anthropogenic, and climatic variables.

| Variable 1 | Variable 2 | Variable 3 | Variable 4 | Variable 5 | Variable 6 | Df | Aic | Δaic |
| --- | --- | --- | --- | --- | --- | --- | --- | --- |
| Aspect | Floor-shelf distance | Isothermality | Annual precipitation |  |  | 4 | 38.29 | **0.00** |
| Aspect | Floor-shelf distance | Distance to town | Isothermality | Annual precipitation |  | 5 | 38.60 | **0.31** |
| Aspect | Cliff wide | Floor-shelf distance | Isothermality | Annual precipitation |  | 5 | 38.88 | **0.59** |
| Aspect | Cliff wide | Floor-shelf distance | Distance to town | Isothermality | Annual precipitation | 6 | 40.12 | **1.83** |
| Aspect | Floor-shelf distance | Distance to town | Annual precipitation |  |  | 4 | 41.18 | > 2 |
| Aspect | Floor-shelf distance | Annual precipitation |  |  |  | 3 | 41.27 | > 2 |
| Aspect | Cliff wide | Floor-shelf distance | Annual precipitation |  |  | 4 | 41.81 | > 2 |
| Aspect | Cliff wide | Floor-shelf distance | Distance to town | Annual precipitation |  | 5 | 42.79 | > 2 |
| Aspect | Cliff wide | Isothermality | Annual precipitation |  |  | 4 | 45.57 | > 2 |
| Aspect | Cliff wide | Distance to town | Isothermality | Annual precipitation |  | 5 | 46.43 | > 2 |
| Cliff wide | Floor-shelf distance | Isothermality | Annual precipitation |  |  | 4 | 47.08 | > 2 |
| Aspect | Isothermality | Annual precipitation |  |  |  | 3 | 47.35 | > 2 |
| Aspect | Cliff wide | Floor-shelf distance | Distance to town | Isothermality |  | 5 | 47.71 | > 2 |
| Aspect | Cliff wide | Floor-shelf distance | Isothermality |  |  | 4 | 48.01 | > 2 |
| Aspect | Distance to town | Isothermality | Annual precipitation |  |  | 4 | 48.11 | > 2 |
| Aspect | Cliff wide | Distance to town | Isothermality |  |  | 4 | 48.47 | > 2 |
| Cliff wide | Floor-shelf distance | Annual precipitation |  |  |  | 3 | 48.60 | > 2 |
| Cliff wide | Floor-shelf distance | Distance to town | Isothermality | Annual precipitation |  | 5 | 48.98 | > 2 |
| Aspect | Cliff wide | Isothermality |  |  |  | 3 | 49.86 | > 2 |
| Cliff wide | Floor-shelf distance | Distance to town | Annual precipitation |  |  | 4 | 50.18 | > 2 |
| Aspect | Floor-shelf distance | Distance to town | Isothermality |  |  | 4 | 50.30 | > 2 |
| Floor-shelf distance | Isothermality | Annual precipitation |  |  |  | 3 | 50.67 | > 2 |
| Aspect | Cliff wide | Floor-shelf distance | Distance to town |  |  | 4 | 51.19 | > 2 |
| Aspect | Floor-shelf distance | Isothermality |  |  |  | 3 | 51.78 | > 2 |
| Aspect | Distance to town | Isothermality |  |  |  | 3 | 52.00 | > 2 |
| Aspect | Cliff wide | Floor-shelf distance |  |  |  | 3 | 52.12 | > 2 |
| Floor-shelf distance | Distance to town | Isothermality | Annual precipitation |  |  | 4 | 52.15 | > 2 |
| Floor-shelf distance | Annual precipitation |  |  |  |  | 2 | 52.77 | > 2 |
| Cliff wide | Floor-shelf distance | Isothermality |  |  |  | 3 | 53.18 | > 2 |
| Floor-shelf distance | Distance to town | Annual precipitation |  |  |  | 3 | 53.60 | > 2 |
| Aspect | Isothermality |  |  |  |  | 2 | 53.94 | > 2 |
| Cliff wide | Floor-shelf distance | Distance to town | Isothermality |  |  | 4 | 54.09 | > 2 |
| Aspect | Floor-shelf distance | Distance to town |  |  |  | 3 | 54.65 | > 2 |
| Aspect | Cliff wide | Distance to town | Annual precipitation |  |  | 4 | 55.14 | > 2 |
| Aspect | Cliff wide | Distance to town |  |  |  | 3 | 55.54 | > 2 |
| Aspect | Cliff wide | Annual precipitation |  |  |  | 3 | 55.85 | > 2 |
| Cliff wide | Floor-shelf distance |  |  |  |  | 2 | 56.07 | > 2 |
| Cliff wide | Floor-shelf distance | Distance to town |  |  |  | 3 | 56.09 | > 2 |
| Cliff wide | Isothermality | Annual precipitation |  |  |  | 3 | 57.24 | > 2 |
| Aspect | Floor-shelf distance |  |  |  |  | 2 | 57.47 | > 2 |
| Cliff wide | Distance to town | Isothermality | Annual precipitation |  |  | 4 | 58.41 | > 2 |
| Cliff wide | Isothermality |  |  |  |  | 2 | 58.53 | > 2 |
| Cliff wide | Distance to town | Isothermality |  |  |  | 3 | 58.58 | > 2 |
| Aspect | Cliff wide |  |  |  |  | 2 | 58.75 | > 2 |
| Aspect | Distance to town | Annual precipitation |  |  |  | 3 | 59.79 | > 2 |
| Floor-shelf distance | Distance to town | Isothermality |  |  |  | 3 | 60.78 | > 2 |
| Floor-shelf distance | Isothermality |  |  |  |  | 2 | 60.79 | > 2 |
| Aspect | Annual precipitation |  |  |  |  | 2 | 61.15 | > 2 |
| Aspect | Distance to town |  |  |  |  | 2 | 61.74 | > 2 |
| Isothermality | Annual precipitation |  |  |  |  | 2 | 63.64 | > 2 |
| Cliff wide | Distance to town |  |  |  |  | 2 | 64.68 | > 2 |
| Floor-shelf distance | Distance to town |  |  |  |  | 2 | 64.81 | > 2 |
| Distance to town | Isothermality | Annual precipitation |  |  |  | 3 | 64.88 | > 2 |
| Cliff wide | Distance to town | Annual precipitation |  |  |  | 3 | 64.89 | > 2 |
| Cliff wide | Annual precipitation |  |  |  |  | 2 | 66.05 | > 2 |
| Aspect |  |  |  |  |  | 1 | 66.25 | > 2 |
| Floor-shelf distance |  |  |  |  |  | 1 | 66.41 | > 2 |
| Distance to town | Isothermality |  |  |  |  | 2 | 66.75 | > 2 |
| Cliff wide |  |  |  |  |  | 1 | 67.11 | > 2 |
| Isothermality |  |  |  |  |  | 1 | 67.17 | > 2 |
| Distance to town | Annual precipitation |  |  |  |  | 2 | 76.94 | > 2 |
| Distance to town |  |  |  |  |  | 1 | 78.41 | > 2 |
| Annual precipitation |  |  |  |  |  | 1 | 78.80 | > 2 |
